# The *Spiritual Supporter Scale* as a New Tool for Assessing Spiritual Care Competencies in Professionals: Design, Validation, and Psychometric Evaluation

**DOI:** 10.1007/s10943-022-01608-3

**Published:** 2022-07-26

**Authors:** Małgorzata Fopka-Kowalczyk, Megan Best, Małgorzata Krajnik

**Affiliations:** 1 Department of Philosophy and Social Sciences, Nicolaus Copernicus University, Toruń, Poland; 2 Institute for Ethics and Society, University of Notre Dame Australia, Sydney, Australia; 3 Department of Palliative Care, Nicolaus Copernicus University in Toruń, Collegium Medicum, Bydgoszcz, Poland

**Keywords:** Spiritual care, Spiritual Supporter Scale, Design, Psychometric evaluation

## Abstract

This study aimed to design, validate and standardize the Spiritual Supporter (SpSup) Scale, a tool designed to assess competency to provide spiritual care including knowledge, sensitivity to spiritual needs and spiritual support skills. This instrument can be used by all those engaged in or training for caregiving roles. The study was conducted in Poland in the Polish language. The SpSup Scale demonstrates high overall reliability (Cronbach’s *α* = 0.88), a satisfactory diagnostic accuracy (0.79), and a satisfactory discriminatory power of the items. Given the psychometric properties of SpSup Scale demonstrated here, the scale is recommended for the assessment of the competency to provide spiritual care in both clinical and research settings in Poland.

## Introduction

Spirituality has long been widely discussed in the caregiving professions as it relates to the provision of comprehensive care, and support for people in difficult situations (Bożek et al., 2020; Chow et al., 2021; Wells-Di et al., 2021; Younkin et al., 2021). While contemporary medical literature increasingly emphasises the need for a holistic approach to support patients and to recognise their somatic suffering in social, mental, and emotional terms (Muszala, 2017; Puchalski et al., 2013; Saunders, 1964), it is also important to consider the individual’s spirituality (Sulmasy, 2002) by using the biopsychosocial–spiritual model of the human being (Balboni et al., 2014; Pawlikowski & Dobrowolska, 2016).

Patients report that they appreciate skill in spiritual care in, and the satisfaction of spiritual needs by, the professionals caring for them (Büssing et al., 2015; O’Callaghan et al., 2019). Spiritual care skills in healthcare professionals contribute to patients’ satisfaction with treatment and care, well-being, and quality of life (Siddall et al., 2015) while reducing anxiety (Hughes et al., 2004) and depression (Bekelman et al., 2007). Patients are able to better cope with disease and have a more positive attitude despite deteriorating health (Brady et al., 1999; Whitford et al., 2008). The relationships between quality of life, coping with disease and receiving spiritual support confirm that spirituality is an essential dimension of patient care (Vandenhoeck, 2013). The importance of spiritual care has been illustrated in diverse groups including: the elderly (Oz et al., 2021), disabled people (Kaye & Raghavan, 2002); as well as oncology (Ben-Arye et al., 2006), psychiatry (Galanter et al., 2011), cardiology (Ozdemir et al., 2021), thoracic (Chen et al., 2021) and HIV-positive patients (Chang et al., 2018; Dalmida et al., 2015). Furthermore, interest in spiritual competencies has also been expressed in the fields of teaching (Epstein, 2018; Harbinson & Bell, 2015), psychotherapy (Mutter et al., 2010; Ren, 2012) and in training for other healthcare professions such as nursing and midwifery (Deluga et al., 2021; McSherry et al., 2021). In summary, gaining competence in and providing spiritual care is important for all professionals who are dealing with people who suffer.

### Need for a New Tool to Assess Competency in Spiritual Care

If spirituality is implicated within the diagnosis and treatment of those experiencing suffering, it is important to ensure that staff are appropriately educated (Lucchetti et al., 2012; Pawlikowski & Dobrowolska, 2016). In order to ensure relevant competencies, a validated tool that allows us to assess and confirm the skill level is needed. A review of the literature reveals many scales for the assessment of spiritual needs (Anandarajah & Hight, 2001; Best et al., 2020; Büssing et al., 2010, 2015; Groves & Klauser, 2009; Maugans, 1996; Neely, 2009; Puchalski, 2002; Ross & McSherry, 2018), and spiritual care competencies, including the Spiritual Care Competency Scale (SCCS) for nurses (Frick et al., 2019; Pastrana et al., 2021), the Spiritual Care Competence Questionnaire (SCCQ) for various professions (Van Leeuwen et al., 2009), and the Servant Leadership and Spirituality Scales (Maglione & Neville, 2021). Tools that examine spirituality or religiousness as a phenomenon are also being developed in Poland (Skowroński & Bartoszewski, 2017). Several tools measuring spirituality in the clinical context were translated and adapted/validated in Poland such as the Self-Description Questionnaire (Heszen-Niejodek & Gruszczyńska, 2004), The Scale of Spiritual Transcendence (Piotrowski et al., 2013), the Brief Religious Coping (RCOPE) Questionnaire (Jarosz, 2011), The Duke University Religion Index-PolDUREL (Dobrowolska et al., 2016), The Spiritual Attitude and Involvement List for nurses (Deluga et al., 2020) or HOPE Scale (Fopka-Kowalczyk et al., 2022). However, none of these scales examine the competencies in diagnosis and spiritual support which we desired to measure. New tools are needed that can assess the impact of teaching spirituality on not only students’ knowledge levels but also their skills and sensitivity in this area, which would ensure students’ competency and effectiveness as future physicians. Our aim was to explore several aspects of spirituality, such as the respondents’ views on spirituality and their ability to recognise suffering and subsequently provide support. We therefore decided to create a new tool when developing, evaluating and implementing the first Polish programme for teaching spirituality to medical students at the Collegium Medicum in Bydgoszcz of the Nicolaus Copernicus University in Toruń, Poland. This article presents the design, validation and standardization of the Spiritual Supporter (SpSup) Scale in Poland, a tool which was developed to assess: competency to provide spiritual care including knowledge, sensitivity to spiritual needs and spiritual support skills, and its standardisation with regard to applicable global standards.

### Definitions of Spirituality

According to Koenig and Al Zaben (2021), ‘the first step in developing a measure of a construct is to define it, as a clear definition will help to assess the quality of items’ (Koenig & Al Zaben, 2021). We therefore based the conceptualization of the tool on the most well-known definitions of spirituality in medicine among Polish students and clinicians. The first was proposed by the European Association for Palliative Care (EAPC) Task Force in 2011 (Nolan et al., 2011) and revised in the EAPC White Paper in 2020 (Best et al., 2020). According to this definition: ‘spirituality is the dynamic dimension of human life that relates to the way persons (individual and community) experience, express and/or seek meaning, purpose and transcendence, and the way they connect to the moment, to self, to others, to nature, to the significant and or the sacred’ (Best et al., 2020, p. 2). The second definition, developed by the Polish Association for Spiritual Care in Medicine (in Polish: Polskie Towarzystwo Opieki Duchowej w Medycynie, PTODM) and adopted as the foundation for the construction of the tool and scale questions presented below, defines spirituality as ‘a dimension of human life that relates to transcendence and other existentially important values’ (PTOMD, 2021). Following the EAPC’s conception, the PTODM identifies similar dimensions of spirituality:

1. Religiousness of a person, especially their relationship with God, personal beliefs, and religious practices, as well as community interaction;
2. Existential quest, especially with regard to: the meaning of life, suffering, and death; issues of personal dignity and personhood; a sense of individual freedom and responsibility, hope and despair, reconciliation and forgiveness, love and joy;
3. Values by which a person lives, especially in relation to oneself and others, work, nature, art and culture, ethical and moral choices, and life at large (PTODM, 2021).

Healthcare professionals should be aware of all these dimensions as a potential source of patients’ coping in the face of death or spiritual suffering.

### Study Objectives

In view of the broad potential application of spiritual care, we decided not to limit ourselves to medical professions but to design a tool that would be useful for all people engaged in or training for caregiving professions, for example medical and healthcare professionals, psychologists, and teachers.

Our objective was the construction and validation of a tool to study:

1. Respondents’ opinions on spirituality and their understanding of their own spirituality;
2. Attitude to spirituality in a relationship with a person in need of care and support;
3. The level of skills necessary to diagnose the spiritual suffering in supported persons;
4. Respondents’ readiness to provide spiritual support to those who suffer.

The proposed scale is intended for students and practitioners in the caregiving professions.

## Methods

### Study Design

The design, development and standardization of the SpSup Scale were carried out according to established standards for the development and psychometric validation of research scales and questionnaires (AERA APA, 2014; Boynton & Greenhalgh, 2004; Brzeziński, 1985; Dogan, 2016; Dufrene & Young, 2014; Koenig & Al Zaben, 2021; Rubacha, 2008; Sousa & Rojjanasrirat, 2011; Wild et al., 2005). Since we conducted our study, Koenig and Al Zaben (2021) have outlined the steps for the development and psychometric validation of a new scale in spirituality measurement and we have followed most of them.

**Fig. 1.**
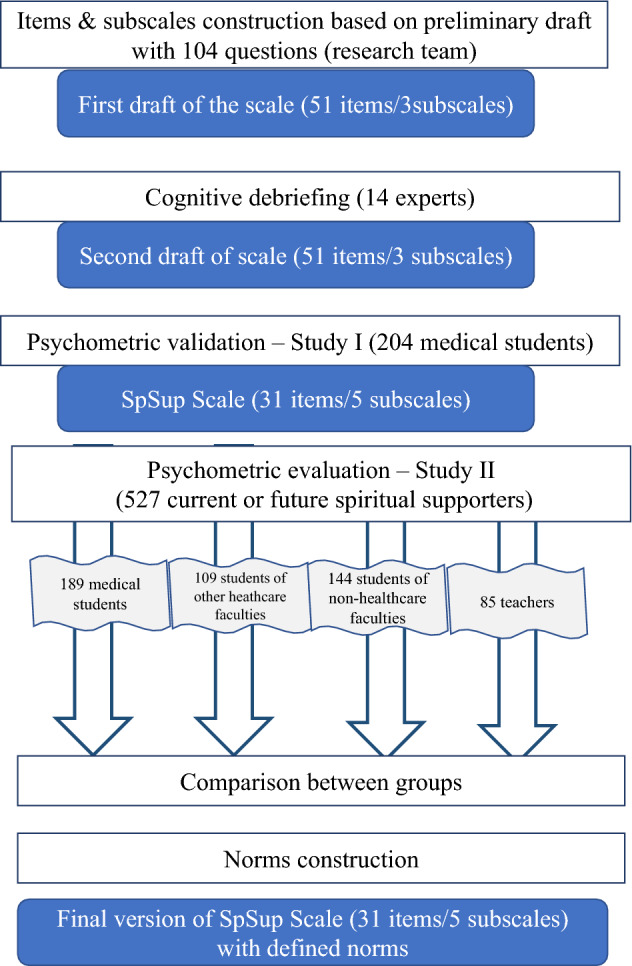
Phases of validation and standardization of SpSup Scale

The stages of scale development are described below in 4 phases: (1) Generation of items; (2) Cognitive debriefing of scale; (3) Validation and standardization—Study I; and (4) Validation and standardization—Study II. Methodology is summarised in Fig. 1, and each phase is explained in full below. All analyses were conducted in SPSS. The next stage of validation is currently underway and will be reported in a future paper. As recommended by Koenig and Al Zaben (2021), the authors will compare the SpSup Scale to existing scales to assess construct validation.

### Participants and Data Collection

The project was approved by the Ethics Committee at Collegium Medicum in Medicum in Bydgoszcz of the Nicolaus Copernicus University in Toruń (number 736/2018) and conducted between 2017 and 2019.

For study I and II of validation and standardization, the questionnaire containing the SpSup Scale was distributed to university students by oral invitation, online or as a printed document. In the case of teachers who participated in study II of validation and standardization, school directors were asked for permission to disseminate the questionnaire in paper format. Completion of the anonymous survey was taken as implied consent.

## Results

### Phase 1: Generation of Items

Potential items for the first draft version of the scale were based on the definitions of spirituality and its dimensions given above (theoretical and definition indicators) (Koenig & Al Zaben, 2021; Rubacha, 2008) and formulated by the research team. The researchers chose those items which corresponded most accurately to the definition of spirituality in medicine. This resulted in a provisional list of 104 items. The first draft of the scale was developed from this list, comprised of 51 items organized as 3 subscales: Me and my beliefs about spirituality (15 items); My spirituality (15 items); My idea of a relationship with a person (as such) experiencing spiritual pain (21 items). The scale included a Likert scale (4 response options from ‘I strongly disagree with this statement’, to ‘I strongly agree’) (Brzeziński, 2004), the instructions and the definition of spirituality.

### Phase 2: Cognitive Debriefing of Scale with 51 Items

According to the literature, cognitive debriefing is used with the target group for whom the scale is prepared, or relevant experts (Sousa & Rojjanasrirat, 2011). The draft questionnaire was therefore assessed by an invited expert panel comprised of 14 members: psychologists (4), physicians (3), nurses (2) and students (5). They were asked to ensure the clarity of questions (“Are items clear and understandable for you?”), and to suggest possible paraphrasing where necessary. They were also asked to provide feedback on the tool regarding its length and usefulness, and the emotions experienced while completing it. During the study, experts were instructed to provide comments about the tool and to propose amendments (“Would you like to change anything in any item?”) (Boynton & Greenhalgh, 2004; Dickie et al., 2018; Patrick et al., 2011; Sousa & Rojjanasrirat, 2011; Wild et al., 2005).

Most of the questions were found to be clear and easy to understand. The structure of the questions was assessed positively. The definition of spirituality included in the scale was also evaluated positively and approved with no reservations. The inclusion of the PTODM’s definition of spirituality in the instructions was vital as in Polish culture the term ‘spirituality’ is frequently perceived as synonymous with religiousness. Without this definition, the study could render inaccurate responses. The experts found four statements incomprehensible and proposed changes to make them clearer (Table 1).

**Table 1:** Cognitive debriefing of the scale

First draft of scale	Suggested changes	Second version of scale
Instructions: the following statements were prepared on the basis of the definition of spirituality	The following statements were prepared based on the definition of spirituality	The following statements were prepared based on the definition of spirituality
In contact with another person, I assume that faith is not an essential element of support for this person	In contact with a patient/person in need of support, I assume that faith is not an important element of this support I do not personally engage in a relationship with another person Faith is not a crucial factor in providing effective support to another person	In a relationship with the patient/person in need of support, not only the body or somatic symptoms but also the actual spiritual suffering and dilemmas are important
When someone complains to me about problems with forgiving, I can recognise it	I can tell when someone in a conversation with me is complaining about problems with forgiving, I try to help them with it When someone says they find it hard to forgive, I can see it	I can tell when someone is suffering on the spiritual level, for example, because they find it hard to forgive

The research team discussed differences in opinion and agreed on the most appropriate versions of the items and wording. As a result, the second draft of the scale was constructed with 51 items and 3 subscales, similar to the first version but with corrected wording and the same Likert scale.

### Phase 3: Study I

The first study of the SpSup Scale was undertaken to establish scale reliability, internal consistency, and discriminatory power of items in a relatively homogenous population.

From 2017 to 2018, participants were recruited from the medical faculties at 2 Polish universities. The sample contained 204 medical students, of whom 127 were female and 67 male (for 10 participants—no data). The median age was 22.99 years (range: 19–30) (Table 2).

**Table 2:** Demographic characteristics of respondents in Study I

Characteristics	*N*	%
*Sex*		
Female	127	62.25
Male	67	32.84
*Age group (years)*		
19–20	7	3.43
21–23	121	59.31
24–26	55	26.96
27–30	9	4.41
*Year of study at the medical study/faculty*		
6 year	26	12.75
5 year	32	15.69
4 year	18	8.82
3 year	110	53.92
2 year	6	2.94
1 year	1	0.49
*No data*		
Sex	10	4.9
Age	12	5.88
Year of study	11	5.39
Total	204	100

#### Psychometric Evaluation of Study I

##### The Internal Consistency of Items and the Initial Reliability of the Scale

In the first step, the items’ discriminatory power was verified to exclude those with a weak correlation with the overall scale score. The results of these calculations are presented in Table 3.

**Table 3:** Discriminatory power of test items—analysis before and after item exclusion

	Before item exclusion			After item exclusion		
Item	M	SD	Discriminatory power	M	SD	Discriminatory power
I1	2.64	0.766	0.572	2.637	0.766	0.565
I2	1.92	0.858	0.608	1.917	0.858	0.608
I3	1.60	0.965	0.357	1.598	0.965	0.384
I4	2.26	0.881	0.178			
I5	1.78	0.990	0.565	1.779	0.990	0.578
I6	1.71	0.843	0.366	1.706	0.843	0.368
I7	2.36	0.834	0.623	2.363	0.834	0.614
I8	2.18	0.988	0.454	2.181	0.988	0.454
I9	1.71	0.812	0.531	1.711	0.812	0.537
I10	2.10	0.882	0.690	2.098	0.882	0.689
I11	2.62	0.743	0.544	2.623	0.743	0.550
I12	1.68	0.942	0.098			
I13	2.44	0.744	0.337	2.441	0.744	0.307
I14	1.88	0.798	0.353	1.882	0.798	0.333
I15	2.24	0.828	0.445	2.240	0.828	0.433
I16	1.97	0.944	0.095			
I17	2.35	0.819	0.517	2.348	0.819	0.519
I18	2.02	0.888	0.570	2.020	0.888	0.578
I19	1.95	0.937	0.612	1.946	0.937	0.620
I20	1.48	1.107	0.522	1.480	1.107	0.570
I21	2.12	0.743	0.115			
I22	2.01	0.944	− 0.155			
I23	1.58	1.152	0.484	1.583	1.152	0.515
I24	1.75	0.770	0.592	1.750	0.770	0.590
I25	2.51	0.705	0.078			
I26	1.87	0.873	0.269	1.873	0.873	0.287
I27	1.92	0.758	0.525	1.922	0.758	0.522
I28	2.30	0.834	0.568	2.304	0.834	0.577
I29	1.87	0.928	0.351	1.873	0.928	0.364
I30	2.37	0.818	0.516	2.373	0.818	0.501
I31	2.18	0.855	0.644	2.181	0.855	0.654
I32	2.13	0.886	0.492	2.132	0.886	0.505
I33	2.43	0.709	0.548	2.431	0.709	0.522
I34	2.06	0.828	0.500	2.059	0.828	0.504
I35	1.52	0.907	0.495	1.520	0.907	0.514
I36	1.81	0.809	0.583	1.814	0.809	0.566
I37	2.18	0.799	0.586	2.176	0.799	0.594
I38	1.89	0.799	0.401	1.892	0.799	0.378
I39	1.58	0.787	0.471	1.578	0.787	0.467
I40	1.51	0.885	0.347	1.510	0.885	0.359
I41	1.93	0.947	0.653	1.926	0.947	0.647
I42	1.75	1.129	0.606	1.745	1.129	0.619
I43	2.31	0.830	0.520	2.314	0.830	0.519
I44	1.84	0.857	0.131			
I45	1.11	1.111	0.446	1.113	1.111	0.475
I46	1.76	0.974	0.388	1.765	0.974	0.417
I47	1.75	0.922	0.370	1.745	0.922	0.364
I48	2.17	0.772	0.527	2.172	0.772	0.534
I49	2.02	0.856	0.577	2.025	0.856	0.569
I50	2.07	0.670	0.379	2.069	0.670	0.367
I51	2.07	0.684	0.293	2.069	0.684	0.278

The initial reliability of the tool, based on Cronbach’s alpha, was 0.929 (95% confidence interval: 0.914–0.942). Following analysis, the statements with Cronbach’s alpha below 0.20 were removed. These questions were excluded from further analyses. Finally, the Cronbach’s alpha was recalculated for all remaining items. The resulting values were satisfactory, with the tool reliability at this stage assessed at 0.940 (95% confidence interval: 0.927–0.951), indicating a very high and satisfactory outcome for our scale.

##### Exploratory Factor Analysis

In the next step, exploratory factor analysis was performed to determine the factor structure of the tool (Table 4). The optimal number of factors was established through parallel analysis (Green et al., 2012; Horn, 1965) to extract the number of factors for which eigenvalues were at least in the 95th percentile of the expected eigenvalue (Green et al., 2012). This method was selected because it is believed to produce the best results of all methods based on eigenvalues (Schmitt, 2011; Zwick & Velicer, 1986). In addition, factor analysis was further justified with the results of Bartlett’s test of sphericity, with correlations between items significantly different from zero (*χ*^2^ [465] = 2964,00; *p* < 0.001). The Kaiser–Meyer–Olkin test confirmed the adequate sample size for factor analysis (KMO = 0.865).

**Table 4:** Results of exploratory factor analysis

	Factor 1	Factor 2	Factor 3	Factor 4	Factor 5	Specific variance
I15	− 0.067	0.132	**0.398**	0.119	0.045	0.727
I24	0.247	− 0.085	**0.429**	0.294	0.118	0.477
I26	0.245	− 0.031	**0.439**	0.007	− 0.257	0.701
I27	0.010	0.029	**0.464**	0.102	0.256	0.585
I33	− 0.204	0.287	**0.468**	− 0.018	0.244	0.499
I36	0.090	0.133	**0.636**	− 0.030	− 0.074	0.496
I38	0.008	0.251	**0.344**	0.180	− 0.278	0.685
I48	0.052	− 0.017	**0.520**	0.169	0.110	0.577
I49	0.007	0.121	**0.572**	0.074	0.055	0.531
I20	**0.865**	0.040	− 0.020	− 0.021	0.048	0.222
I23	**0.852**	0.155	− 0.124	0.037	− 0.073	0.254
I42	**0.682**	− 0.067	0.073	0.099	0.240	0.354
I45	**0.750**	− 0.055	0.125	− 0.087	0.012	0.409
I46	**0.491**	− 0.063	0.305	0.024	− 0.133	0.621
I1	− 0.054	**0.466**	0.230	0.162	0.021	0.562
I7	0.033	**0.732**	− 0.016	0.009	0.137	0.354
I10	0.255	**0.454**	0.057	0.017	0.178	0.493
I13	− 0.067	**0.672**	− 0.153	− 0.008	− 0.049	0.652
I19	0.233	**0.515**	0.120	− 0.030	0.033	0.512
I28	0.018	**0.419**	0.278	0.027	0.051	0.599
I31	0.133	**0.536**	0.169	− 0.035	0.082	0.491
I34	0.172	**0.489**	0.022	0.016	− 0.006	0.665
I37	0.042	**0.450**	0.197	0.144	0.081	0.541
I43	0.074	**0.305**	0.114	0.263	0.036	0.688
I17	− 0.009	0.239	0.140	− 0.051	**0.476**	0.568
I29	0.080	0.026	− 0.132	0.049	**0.683**	0.506
I32	0.025	0.106	0.074	0.032	**0.694**	0.386
I35	0.254	0.004	0.182	0.019	**0.378**	0.644
I39	0.059	0.186	0.156	**0.451**	− 0.099	0.631
I50	− 0.061	− 0.019	0.046	**0.851**	0.005	0.266
I51	0.029	− 0.013	− 0.119	**0.794**	0.010	0.419

Items included in the factor framework are marked in bold

The analysis showed five factors that explained 48% of the variance in all items. Some items did not load on any of the corresponding factors or presented high factor loadings on more than one latent variable. The final factor solution is shown in Table 5. Given the expected (and existing) correlations between the factors, rotated factor loadings are presented (oblimin rotation).

**Table 5:** Correlations between factors

	Factor 1	Factor 2	Factor 3	Factor 4	Factor 5
Factor 1	–	0.362	0.365	0.195	0.279
Factor 2		–	0.539	0.271	0.501
Factor 3			–	0.452	0.305
Factor 4				–	0.256
Factor 5					–

##### Scale Reliability

In order to check the reliability of the scale, Cronbach’s alpha (with 95% confidence interval) and McDonald’s omega were calculated (AERA APA, 2014). The results are presented in Table 6.

**Table 6:** Reliability of the tool and subscales

	McDonald’s *Ω*	Cronbach’s *α*	Cronbach’s *α* 95% CI		Average inter-item correlation
			Lower threshold	Upper threshold	
Scale overall	0.922	0.920	0.903	0.935	0.274
Factor 1	0.875	0.873	0.843	0.898	0.573
Factor 2	0.870	0.869	0.841	0.894	0.398
Factor 3	0.825	0.819	0.779	0.854	0.338
Factor 4	0.760	0.738	0.668	0.794	0.495
Factor 5	0.752	0.743	0.680	0.796	0.421

The results for the scale points and the 95% confidence interval indicated a high level of internal consistency for the scale overall and individual subscales. Scales 4 and 5 featured a slightly lower, albeit still acceptable, level of reliability. The discriminatory power of the items was re-estimated with regards to the overall score and individual subscales (Table 7). All indicators exceeded the value of 0.20 and can therefore be considered satisfactory.

**Table 7:** Discriminant power of items in the version of the tool in Study I

	Item	M	SD	Discriminant power
Scale overall	I15	2.240	0.828	0.409
	I24	1.750	0.770	0.607
	I26	1.873	0.873	0.314
	I27	1.922	0.758	0.530
	I33	2.431	0.709	0.527
	I36	1.814	0.809	0.555
	I38	1.892	0.799	0.370
	I48	2.172	0.772	0.527
	I49	2.025	0.856	0.564
	I20	1.480	1.107	0.577
	I23	1.583	1.152	0.529
	I42	1.745	1.129	0.618
	I45	1.113	1.111	0.478
	I46	1.765	0.974	0.437
	I1	2.637	0.766	0.556
	I7	2.363	0.834	0.612
	I10	2.098	0.882	0.641
	I13	2.441	0.744	0.289
	I19	1.946	0.937	0.616
	I28	2.304	0.834	0.562
	I31	2.181	0.855	0.634
	I34	2.059	0.828	0.499
	I37	2.176	0.799	0.612
	I43	2.314	0.830	0.502
	I17	2.348	0.819	0.487
	I29	1.873	0.928	0.334
	I32	2.132	0.886	0.513
	I35	1.520	0.907	0.506
	I39	1.578	0.787	0.451
	I50	2.069	0.670	0.372
	I51	2.069	0.684	0.302
Factor 1	I20	1.480	1.107	0.799
	I23	1.583	1.152	0.742
	I42	1.745	1.129	0.726
	I45	1.113	1.111	0.725
	I46	1.765	0.974	0.509
Factor 2	I1	2.637	0.766	0.574
	I7	2.363	0.834	0.700
	I10	2.098	0.882	0.628
	I13	2.441	0.744	0.438
	I19	1.946	0.937	0.634
	I28	2.304	0.834	0.579
	I31	2.181	0.855	0.656
	I34	2.059	0.828	0.543
	I37	2.176	0.799	0.623
	I43	2.314	0.830	0.474
Factor 3	I15	2.240	0.828	0.443
	I24	1.750	0.770	0.606
	I26	1.873	0.873	0.354
	I27	1.922	0.758	0.538
	I33	2.431	0.709	0.510
	I36	1.814	0.809	0.643
	I38	1.892	0.799	0.409
	I48	2.172	0.772	0.571
	I49	2.025	0.856	0.622
Factor 4	I39	1.578	0.787	0.463
	I50	2.069	0.670	0.638
	I51	2.069	0.684	0.605
Factor 5	I17	2.348	0.819	0.525
	I29	1.873	0.928	0.540
	I32	2.132	0.886	0.647
	I35	1.520	0.907	0.442

The final outcome of the first standardisation performed as Study I was a questionnaire consisting of 31 questions organised into five subscales:

1. Attitude to prayer (5 items).
2. Beliefs regarding spirituality (10 items).
3. Spirituality in relation to one’s own suffering and the suffering of others (9 items).
4. Sensitivity to the suffering of others (3 items).
5. Attitude to community (4 items).

### Phase 4: Study II

The second study of the SpSup Scale was undertaken to establish the psychometric properties of the scale (e.g. scale reliability, internal consistency, discriminatory power of items, exploratory factor analysis) and was performed on a larger and more diverse population of respondents. In addition, the comparison of psychometric factors between different groups of participants was performed. At the end of scale standardisation, the final norms were defined, leading to the final version of SpSup Scale.

#### Characteristics of the Sample

The sample collected from 2018 to 2020 contained 527 participants who were working or preparing to work as professional caregivers: medical students, students of other healthcare faculties, students of non-healthcare faculties and teachers) of whom 416 (79%) were female and 96 (18.22%) male (no data: *n* = 15, 2.85%). The median age was 25.76 years, with age range 19–70 years.

Four comparative groups were distinguished based on occupational affiliations. As a result, the following groups were studied: teachers (*n* = 85; 16.13%), medical students (*n* = 189; 35.86%), students of other healthcare faculties (*n* = 109; 20.68%), and students of non-healthcare faculties (*n* = 144; 27.32%).

In the teacher group, most of the respondents were female (*n* = 54; 63.53%). The average age in this subgroup was 46.55 years (range 24–70 years) with average professional experience of 22.92 years (SD = 7.87; range 4–45 years). In the group of medical students, the mean age of the respondents was 24.28 years (range 22–28) with a majority of women (*n* = 125; 66.14%). At the time of the study, all students in this group were in the fifth year of study. In the group of students of other healthcare faculties, most students were in their third year of bachelor level study (*n* = 78; 71.56%), while the remainder were second year students of master level study. The group was dominated by women (*n* = 104; 95.41%). The average age in this subgroup was 22.34 years (range 21–29 years). The group of non-healthcare students was dominated by first-year and second year students of bachelor level study (*n* = 90; 62.50%; and *n* = 12; 8.33%, respectively), while the remainder were first year students of master level study. The average age in this subgroup was 20.77 years (range 19–25 years). More information about the demographic characteristics in Study II is presented in Table 8.

**Table 8:** Demographic characteristics of respondents in Study 2

Characteristics	Participants		*N*	%
SEX	Medical students	Female	125	66.14
		Male	64	33.86
		All	189	100
	Other healthcare faculties	Female	104	95.41
		Male	5	13.76
		All	109	100
	Non-healthcare faculties	Female	133	92.36
		Male	8	5,56
		n.d	3	2.08
		All	144	100
	Teachers	Female	54	63.53
		Male	19	22.35
		n.d	12	14.12
		All	85	100
AGE (years)	Medical students	22–23	62	32.8
		24–25	92	48.68
		26–28	35	18.52
		All	189	100
	Other healthcare faculties	21	44	40.37
		22–23	44	40.37
		24–25	17	16.6
		26–29	4	3.67
		All	109	100
	Non-health care faculties	19	44	30.56
		20–21	50	34.72
		22–23	43	29.86
		24–25	7	4.86
		All	144	100
	Teachers	24–30	4	4.71
		30–40	12	14.12
		40–50	29	34.12
		More than 50	21	24.7
		n.d	19	22.35
		All	85	100
YEAR OF STUDY AT UNIVERSITY	Medical students	5 year of study	189	100
	Other healthcare faculties	3 year of bachelor study	78	71.56
		2 year of master level study	31	28.44
		All	109	100
	Non-health care faculties	1 year bachelor study	90	62.5
		II year of bachelor study	12	8.33
		I year of master level study	42	29.2
		All	144	100
YEARS OF WORKING	Teachers	To 10	5	5.88
		Nov-20	21	24.71
		21–30	37	43.53
		More than 30	5	5.88
		n.d	17	20
		All	85	100
FACULTY/PROFESSIONS	Medical students	Physicians	189	100
	Other healthcare faculties	Electroradiology	1	0.92
		Nursing	78	71.56
		Widwifery	30	27.2
		All	109	100
	Non-healthcare faculties	Pedagogy	100	69.44
		Social work	44	30.56
		All	144	100
	Teachers		85	100
TOTAL			527	100

#### Psychometric Evaluation of Study II

##### Factor Structure

The theoretical structure developed in Study I was tested using confirmatory factor analysis (CFA). It allowed us to verify the adequacy of the five-factor model. Given the ordinal measurement level of the scale and the significant skewness and kurtosis of some items (skewness above ± 2.0 was found in Item 1; kurtosis above ± 2.0 was found in items 1, 2, 4, 25), the diagonally weighted least squares (DWLS) method was used for the model estimation.

The five-factor model turned out to demonstrate a satisfactory model fit: *χ*^2^(424) = 762.01; *p* < 0.001; Root mean square error (RMSEA) = 0.04 [90% CI: 0.034; 0.043]; CFI = 0.97; TLI = 0.96, GFI = 0.96. The standardised values of covariance between factors are presented in Table 9.

**Table 9:** Factor covariance in the Spiritual Supporter (SpSup) Scale—standardised covariance values

		1	2	3	4	5
1	Spirituality in relation to one’s own suffering and the suffering of others					
2	Attitude to prayer	0.41***	–			
3	Beliefs regarding spirituality	0.55***	0.49***	–		
4	Attitude to community	0.51***	0.44***	0.66***	–	
5	Sensitivity to the suffering of others	0.72***	0.24***	0.46***	0.35***	–

^***^p < 0.001

The results suggested that the identified latent factors represented a significant part of the ‘shared’ variance in many cases. In view of this, it was necessary to verify whether the variance was sufficiently significant to provide the basis for isolating the second-order factor to explain the covariance of the first-order factors. To this end, the CFA was performed again to test the fit of the hierarchical model with one second-order factor and five first-order factors. The model demonstrated an acceptable model fit: *χ*^2^ (429) = 879.09; *p* < 0.001; RMSEA = 0.05 [90% CI 0.040; 0.049]; CFI = 0.95; TLI = 0.95, GFI = 0.95. In both cases, factor loadings for individual items were generally satisfactory and statistically significant. Item 11 was an exception as its fully standardised factor loading was 0.08 and 0.09 for Models 1 and 2, respectively. Nevertheless, it was statistically significant. The exact values ​​of the fully standardised factor loadings for both models are presented in Table 10.

**Table 10:** Non-standardised and fully standardised factor loadings for models tested in confirmatory factor analysis (CFA)

Factor	Item	Model 1: Five first-order factors				Model 2: Five first-order factors and one second-order factor			
		λ	*Z*	*p*	*λ*_stand_	λ	*Z*	*p*	*λ*_stand_
1	P5	0.23	13.31	< 0.001	0.29	0.16	11.21	< 0.001	0.29
	P10	0.32	19.59	< 0.001	0.47	0.21	14.12	< 0.001	0.46
	P11	0.07	3.73	< 0.001	0.08	0.052	3.79	< 0.001	0.09
	P12	0.36	23.70	< 0.001	0.61	0.24	15.46	< 0.001	0.60
	P17	0.31	22.27	< 0.001	0.55	0.21	15.31	< 0.001	0.55
	P20	0.49	25.40	< 0.001	0.66	0.33	16.02	< 0.001	0.66
	P22	0.17	10.37	< 0.001	0.25	0.11	8.96	< 0.001	0.24
	P28	0.24	12.23	< 0.001	0.28	0.16	10.57	< 0.001	0.28
	P29	0.43	22.99	< 0.001	0.60	0.30	15.46	< 0.001	0.61
2	P8	0.91	35.62	< 0.001	0.85	0.75	30.44	< 0.001	0.85
	P9	0.97	36.47	< 0.001	0.89	0.80	30.89	< 0.001	0.89
	P24	0.80	33.09	< 0.001	0.83	0.66	28.80	< 0.001	0.83
	P26	67	32.70	< 0.001	0.73	0.56	28.64	< 0.001	0.74
	P27	0.35	16.18	< 0.001	0.38	0.29	15.70	< 0.001	0.38
3	P1	0.38	21.57	< 0.001	0.55	0.22	13.50	< 0.001	0.55
	P2	0.41	23.09	< 0.001	0.61	0.24	13.80	< 0.001	0.61
	P3	0.41	24.44	< 0.001	0.60	0.24	14.15	< 0.001	0.60
	P4	0.27	19.31	< 0.001	0.46	0.15	12.87	< 0.001	0.46
	P7	0.54	29.10	< 0.001	0.69	0.31	15.08	< 0.001	0.69
	P13	0.40	24.85	< 0.001	0.60	0.23	14.39	< 0.001	0.60
	P15	0.54	30.56	< 0.001	0.73	0.31	15.29	< 0.001	0.73
	P18	0.23	15.90	< 0.001	0.33	0.14	11.98	< 0.001	0.33
	P21	0.42	26.09	< 0.001	0.65	0.24	14.51	< 0.001	0.65
	P25	0.35	24.29	< 0.001	0.55	0.20	14.39	< 0.001	0.55
4	P6	0.30	16.51	< 0.001	0.47	0.20	10.98	< 0.001	0.47
	P14	0.50	20.43	< 0.001	0.60	0.33	11.86	< 0.001	0.60
	P16	0.48	21.30	< 0.001	0.66	0.32	11.97	< 0.001	0.66
	P19	0.65	23.59	< 0.001	0.71	0.43	12.38	< 0.001	0.71
5	P23	0.49	19.82	< 0.001	0.72	0.40	14.37	< 0.001	0.73
	P30	0.44	18.73	< 0.001	0.69	0.35	13.85	< 0.001	0.69
	P31	0.39	17.22	< 0.001	0.60	0.31	13.14	< 0.001	0.59

Given the results, the theoretical validity can be assumed to have been confirmed in terms of factor stability. Furthermore, the acceptable fit of the hierarchical model with the second-order factor also indicates that, next to five specific dimensions of spirituality, a primary dimension can be distinguished, being the overall spiritual awareness.

##### Scale Reliability and Discriminatory Power of Items

The mean scores, standard deviations, and other descriptive statistics for the dimensions of spirituality and overall test score are presented in Table 11. This table also shows the reliability levels for individual measurements. Reliability was assessed using Cronbach’s alpha and McDonald’s omega (AERA APA, 2014). The last of the measures was calculated due to the lack of strict unidimensionality in the analysed test.

**Table 11:** Descriptive statistics and scale reliability

Skala		Descriptive statistics				Reliability			
		*M*	SD	Min	Max	McDonald’s *ω*	Cronbach’s *α*	Discriminant power (min; max)	Average inter-item correlation
1	Spirituality in relation to one’s own suffering and the suffering of others	17.96	3.40	6	26	0.65	0.65	0.15; 0.48	0.19
2	Attitude to prayer	7.90	3.95	0	15	0.87	0.85	0.36; 0.81	0.53
3	Beliefs regarding spirituality	24.03	4.41	3	30	0.84	0.84	0.30; 0.67	0.35
4	Attitude to communion	8.58	2.33	0	12	0.74	0.73	0.37; 0.62	0.42
5	Sensitivity to the suffering of others	5.80	1.59	0	9	0.74	0.73	0.44; 0.63	0.48
6	Overall test score	64.27	11.11	30	91	0.88	0.88	0.08; 0.60	0.20

Nearly all subscales demonstrated a satisfactory level of reliability, with the highest observed for *Attitude to prayer*. Reliability was also satisfactory for the overall spirituality level. Only the *Spirituality in relation to one’s own suffering* and the *Suffering of others* subscales were characterised by a borderline reliability level (above 0.60), which was attributed to a lower mean correlation between items (0.19). Nevertheless, all subscales should be considered to be potentially useful.

##### Differences in Spirituality Between Groups According to Sex, Profession and Age

The dimensions of spirituality were tested for possible sex-specific differences. Given the considerable differences in the size of both groups and the lack of normal distributions for the tested variables, the groups were compared using the Mann–Whitney U test.

The analysis showed no statistically significant sex-specific differences for *Attitude to prayer*; *Beliefs regarding spirituality*; and *Sensitivity to suffering*. Minor differences (small effect size) between females and males were observed for *Spirituality in relation to one’s own suffering*; *Suffering of others*; and *Attitude to community*. Women demonstrated higher scores on these scales. They also presented a higher level of overall spirituality, with a small effect size of differences for men. The spirituality dimensions were also tested for possible differences among the four identified professional groups.

To this end, a one-way analysis of variance (ANOVA) was used with ω^2^ values to measure the effect size. The analysis showed no statistically significant differences for *Attitude to prayer*; *Spirituality in relation to one’s own suffering*; and *Suffering of others*. However, minor differences (small effect size) among the groups were observed for *Attitude to community*; *Sensitivity to the suffering of others;* and overall spirituality level.

Tukey’s test was used for pairwise post hoc testing. It revealed statistically significant differences for *Attitude to community* only in the comparison of students of other healthcare faculties with students of non-healthcare faculties: *t* = 3.22; *d* = 0.43; *p* = 0.007. Cohen’s d showed a medium effect size for differences. The scores for *Attitude to community* were higher for students of other healthcare faculties compared with students of non-healthcare faculties (*M* = 9.09; SD = 2.12; and *M* = 8.15; SD = 2.27, respectively). No differences were observed between other groups for this dimension of spirituality.

The pairwise comparisons revealed no statistically significant differences for *Sensitivity to the suffering of others*. However, a comparison between future physicians (medical students) and students of non-healthcare faculties showed a trend towards statistical significance: *t* = 2.40; *d* = 0.25; *p* = 0.078. A similar trend was observed when comparing future physicians with teachers: *t* =  − 2.39; *d* =  − 0.31; *p* = 0.081. The effect size for both differences was medium. The scores for *Sensitivity to the suffering of others* were higher for medical students compared with students of non-healthcare faculties and teachers (*M* = 6.01; SD = 1.62 for future physicians; *M* = 5.59; SD = 1.72 for students of non-healthcare faculties; and *M* = 5.52; SD = 1.52 for teachers). No differences were observed between other groups for this dimension of spirituality.

Regarding the overall level of spirituality, statistically significant differences were observed only when comparing students of other healthcare faculties with students of non-healthcare faculties: *t* = 3.61; *d* = 0.48; *p* = 0.002. The effect size for the differences was moderate. The former group had higher scores for the overall level of spirituality compared with the latter group (*M* = 66.92; SD = 9.62; and *M* = 61.88; SD = 11.27, respectively). No differences were observed between other groups in this dimension of spirituality.

We investigated whether the respective dimensions of spirituality were related to respondents’ age and seniority (in this case, correlations were calculated only for the group of teachers in which this variable was measured). Given the significant sample size, the Pearson correlation coefficient was used. The analysis showed no statistically significant relationships between spirituality and its dimensions and the demographic variables of age and seniority. In terms of the factors, the only (very weak) correlation was found between *Attitude to prayer* and seniority (*r* = 0.09; *p* = 0.047).

##### Diagnostic Accuracy

To estimate the diagnostic accuracy of a test, one needs to compare the results obtained in a tested group of respondents with an external criterion that allows us to assess the same variable as the one measured by that test. In our case, the external criterion was defined as the respondents’ behaviour, for instance, their opinion regarding spiritual support and care in a specific situation (task). To this end, 43 subjects were asked to complete the scale, while the results were calculated using Yule’s formula. The result was 0.79, with the estimated significance level *ϰ*^2^ = 27.51.

The chi-square critical value was from 3.841, ∞, which means that the obtained result *ϰ*^2^ falls within this range. The result can therefore be assumed to be statistically significant at *p* < 0.05. Consequently, the relationship between the respondents’ perception of their ability to perform a given task and the SpSup Scale score was found to be true, with the type I error probability of 0.001 (one in 1000) or less.

##### Sten Scores and the Key for the Scale Calculations

The final step in the development of the proposed questionnaire was to establish a standardised scale for the calculation of the scale scores. Given that no significant differences were found among groups in terms of the dimensions of spirituality, common standards were adopted for all respondents using Sten scores. Scores within Sten 1–2 were defined as very low, 3–4 as low, 5–6 as medium, 7–8 as high, and 9–10 as very high.

## Discussion

Training courses for people in caregiving professions, such as physicians, nurses, midwives, psychologists, pedagogists, teachers, chaplains and other helpers, focus on improvement in skills, an outcome which requires evaluation (Cortés-Rodríguez et al., 2022; Moore et al., 2018; Puchalski et al., 2021). As our university was the first in Poland to introduce spirituality into the medical curriculum, we wanted to develop a scale to assess the outcomes of this programme. A literature review showed that, despite the availability of several spiritual care tools, none of them captured the variables of interest to us (Deluga et al., 2020; Dobrowolska et al., 2016; Heszen-Niejodek & Gruszczyńska, 2004; Jarosz, 2011; Piotrowski et al., 2013). Furthermore, we wanted a scale that was relevant beyond healthcare. After methodological consultations, the target group of the SpSup Scale was extended, and the scale can now be used to test any adult working in or preparing for a caregiving profession. Results of the validation and standardization of our tool and the obtained psychometric values are highly satisfactory. It is worth highlighting the overall high reliability of the scale (Cronbach’s *α* = 0.88) and subscales (1 = 0.65; 2 = 0.85; 3 = 0.84; 4 = 0.73; 5 = 0.73), a satisfactory diagnostic accuracy (0.79, with the estimated significance level *ϰ*^2^ = 27.51), and a satisfactory discrimination index. Construct validation of the SpSup Scale is currently underway through correlation with similar scales, as recommended by Koenig and Al Zaben (2021, pp. 3475–3476).

As such, the SpSup Scale is recommended for the assessment of spiritual care, in both clinical and research settings, with regards to the following components: (1) Respondents’ opinions on spirituality and their understanding of their own spirituality; (2) Attitude to spirituality in a relationship with a person in need of care and support; (3) The level of skills necessary to diagnose the spiritual suffering in supported persons; and (4) Respondents’ readiness to provide spiritual support to those who suffer.

### Study Limitations

This study has some limitations. The questionnaire’s format as it stands may be too long for everyday use. Future studies should investigate whether a shorter version of the scale could be created. In addition, future research studies should replicate the present study using large cohorts to establish correlation between SpSup Scale and factors such as personality or emotional intelligence. We also believe that cross-comparing findings among multiple professional domains would reveal insightful and useful findings.

## Conclusions

Supporting others requires many competencies and skills from professionals. In addition to knowledge, experience and technical skills directly related to the specific profession, people looking for support are increasingly expecting interpersonal competencies in their caregivers, including those related to spiritual support. Regardless of their belief system, a suffering person wants to be treated not only by a specialist qualified in a specific field, but also by a fellow human being capable of showing concern, recognising emotions, talking, and offering help.

Many universities are implementing programmes for the development of interpersonal attitudes and other qualifications necessary for specialists to show multi-level support suited to clients’/patients’ needs. To evaluate the effect of such training courses, it is necessary to have appropriate measures. The Polish version of the SpSup Scale has been constructed as an instrument for measuring spiritual competencies among professionals. Considering the good psychometric properties of the tool, its use is recommended for the assessment of spiritual care and support, along with their components, in both clinical and research settings.

## Appendix 1: Polish version

**Kwestionariusz Troski Duchowej – KTD** (Małgorzata Fopka-Kowalczyk, Małgorzata Krajnik)

**INSTRUKCJA:** Prezentujemy Państwu kwestionariusz dotyczący przekonań na temat troski duchowej. Twierdzenia zostały stworzone w oparciu o definicję duchowości przedstawioną poniżej. Proszę uważnie przeczytać definicję oraz każde twierdzenie i postarać się odpowiedzieć na każde z nich według podanego wzoru:

- 1. Zdecydowanie nie zgadzam się z danym twierdzeniem
- 2. Raczej nie zgadzam się z twierdzeniem
- 3. Raczej zgadzam się z twierdzeniem
- 4. Zdecydowanie zgadzam się z danym twierdzeniem

Definicja duchowości Polskiego Towarzystwa Opieki Duchowej w Medycynie (PTODM):^1^

Duchowość to wymiar ludzkiego życia stanowiący odniesienie do transcendencji i innych wartości egzystencjalnie ważnych

Wymiary duchowości:

1. religijność człowieka, zwłaszcza jego relacje z Bogiem, a także zwyczaje i praktyki oraz życie wspólnotowe
2. poszukiwania egzystencjalne odnoszące się szczególnie

– do sensu życia, cierpienia i śmierci oraz do odpowiedzi na pytanie o własną godność i o to kim się jest jako osoba

– do sfery wolności i odpowiedzialności, nadziei i rozpaczy, pojednania i przebaczenia, miłości i radości

3) wartości, którymi żyje człowiek, zwłaszcza jego relacje z samym sobą i z innymi ludźmi, stosunek do pracy, natury, sztuki i kultury, jego wybory w sferze moralności i etyki oraz „samo życie”.

Podstawowe dane:

- 1.Wiek:
- 2. Płeć: K M
- 3. Staż pracy/zawód wykonywany/rok i kierunek studiów:

**Table:**

Lp	Twierdzenie	Zdecyd. nie	Raczej nie	Raczej tak	Zdecyd. tak
1	Duchowość jest czymś więcej niż tylko przynależnością do instytucji kościelnej	1	2	3	4
2	Duchowość przejawia się również w relacjach z drugim człowiekiem	1	2	3	4
3	Przebaczenie jest jednym z wymiarów duchowości	1	2	3	4
4	Duchowość związana jest ze sferą życia wewnętrznego człowieka	1	2	3	4
5	Gdy ktoś cierpi, sprawia mi trudność okazywanie współczucia	1	2	3	4
6	Sens w moim życiu nadaje obecność innych bliskich mi osób	1	2	3	4
7	Uważam, że w pracy z drugim człowiekiem powinienem brać pod uwagę także sprawy duchowe doświadczane przez tą osobę	1	2	3	4
8	Relacja z Bogiem jest treścią mojego życia duchowego	1	2	3	4
9	Wierzę, że modlitwa pomaga mi w życiu	1	2	3	4
10	Umiem być całkowicie obecny przy cierpiącym człowieku	1	2	3	4
11	Wiem, że moje doświadczenia życiowe utrudniają mi zachowanie równowagi i spokoju duchowego	1	2	3	4
12	Próbuję być obecny przy człowieku, który doświadcza beznadziei	1	2	3	4
13	Każdy pomagający powinien umieć zadbać w sposób całościowy o pacjenta/osobę potrzebującą wsparcia, a nie tylko o jego ciało	1	2	3	4
14	Potrzebuję innych ludzi w sytuacjach, gdy nie znajduje sensu w życiu	1	2	3	4
15	Myślę, że rozmowa o problemach duchowych wzmacnia człowieka i daje mu siłę do radzenia sobie z przeciwnościami takimi jak na przykład choroba czy kryzys	1	2	3	4
16	Gdy tracę nadzieję, to obecność drugiego człowieka pomaga mi przetrwać i odnaleźć nadzieję na nowo	1	2	3	4
17	Gdy ktoś chce porozmawiać o tym, co jest dla niego ważne w życiu, chętnie go wysłuchuje	1	2	3	4
18	Uważam, że koncentracja na problemach duchowych odbiera nadzieję	1	2	3	4
19	Bycie członkiem bliskiej mi grupy, społeczności czy wspólnoty daje mi silę w życiu	1	2	3	4
20	Umiem rozmawiać z drugim człowiekiem o jego trudnościach duchowych	1	2	3	4
21	W relacji z pacjentem/osobą potrzebującą wsparcia ważne jest nie tylko ciało i występujące dolegliwości somatyczne, ale też przeżywane cierpienie czy dylematy duchowe	1	2	3	4
22	Umiem zadbać o własne wnętrze duchowe	1	2	3	4
23	Potrafię rozpoznać, gdy ktoś przeżywa cierpienie duchowe, np. z powodu trudności z przebaczeniem	1	2	3	4
24	Gdy ktoś mnie poprosi, modlę się z tą osobą	1	2	3	4
25	Uważam, że to ważne, aby nadawać w życiu znaczenie temu, co się robi	1	2	3	4
26	Czasem sam proponuję modlitwę	1	2	3	4
27	Nie wiem, jak się zachować, gdy ktoś oczekuje ode mnie modlitwy	1	2	3	4
28	Uciekam od zwierzeń	1	2	3	4
29	Mam dość swoich problemów, aby jeszcze zajmować się bólem duchowym innych osób	1	2	3	4
30	Czuję, gdy ktoś cierpi i doświadcza trudnych chwil	1	2	3	4
31	Umiem rozpoznać cierpienie drugiego człowieka poprzez jego mowę ciała	1	2	3	4

## Appendix 2: English version

***Spiritual Supporter Scale*****—SPSup Scale** (Małgorzata Fopka-Kowalczyk, Małgorzata Krajnik)

SpSup—translated from the original Polish version of Spiritual Supporter Scale, not yet validated in English.

**INSTRUCTIONS:** This questionnaire examines beliefs about spiritual care provided to a suffering person who needs spiritual support. The following statements were prepared based on the definition of spirituality presented below. Please read the definition and all statements carefully and try to respond to each statement using the following formula:

1. I strongly disagree with this statement
2. I disagree with this statement
3. I agree with this statement
4. I strongly agree with this statement

Definition of spirituality proposed by Polskie Towarzystwo Opieki Duchowej w Medycynie/Polish Association for Spiritual Care in Medicine (PTODM):^2^

Spirituality is a dimension of human life that relates to transcendence and other existentially important values.

Dimensions of spirituality:

- 1) Religiousness of a person, especially their relationship with God, personal beliefs, and religious practices, as well as community interaction;
- 2) Existential quest, especially regarding:
- – the meaning of life, suffering, and death, issues of own dignity, who one actually is as a person;
- – a sense of individual freedom and responsibility, hope and despair, reconciliation and forgiveness, love and joy.
- 3) Values by which a person lives, especially with regards to oneself and others, work, nature, art and culture, ethical and moral choices, and life at large.

Basic data:

- 1. Age:
- 2. Sex: F / M
- 3. Years professional experience/profession/year and field of study

**Table:**

No	Items	Strongly disagree	Disagree	Agree	Strongly agree
1	Spirituality is something more than being part of organised religion	1	2	3	4
2	Spirituality can be manifest through relationships with other people	1	2	3	4
3	Forgiveness is one of the dimensions of spirituality	1	2	3	4
4	Spirituality is related to the sphere of inner human life	1	2	3	4
5	When someone is suffering, I find it difficult to show compassion	1	2	3	4
6	The presence of loved ones gives my life meaning	1	2	3	4
7	When working with another person, I should consider the spiritual issues experienced by that person	1	2	3	4
8	My relationship with God is the essence of my spiritual life	1	2	3	4
9	I believe prayer helps me in life	1	2	3	4
10	I know how to be truly present with a suffering person	1	2	3	4
11	I know that my life experiences make it difficult for me to maintain balance and spiritual peace	1	2	3	4
12	I try to be present with a person who is experiencing the feeling of hopelessness	1	2	3	4
13	Each helper should be able to provide holistic care to the patient/person in need of support rather than only care for the physical aspects	1	2	3	4
14	I need other people to be present in situations when I see no meaning in my life	1	2	3	4
15	I think that talking about spiritual problems makes people stronger and gives them the power to cope with adversities such as illness or crisis	1	2	3	4
16	When I lose hope, the presence of another person helps me get through this and find hope again	1	2	3	4
17	When someone wants to talk about things that are important to them in life, I am eager to listen	1	2	3	4
18	I think that focusing on spiritual problems takes hope away	1	2	3	4
19	Being a member of a group or a community gives me strength in my life	1	2	3	4
20	I can talk to another person about their spiritual difficulties	1	2	3	4
21	In a relationship with a patient/person in need of support, not only the body or physical symptoms but also the actual spiritual suffering and dilemmas are important	1	2	3	4
22	I can nurture my own spirituality	1	2	3	4
23	I can tell when someone is suffering on the spiritual level, for example, because they find it hard to forgive	1	2	3	4
24	When a person asks me to pray with them, I do it	1	2	3	4
25	I think it is important to give meaning to what you do in life	1	2	3	4
26	Sometimes I suggest to patients/clients that we pray together	1	2	3	4
27	I don’t know what to do when someone expects me to pray with them	1	2	3	4
28	I avoid confiding in other people	1	2	3	4
29	I have enough of my own problems to deal with other people’s spiritual pain	1	2	3	4
30	I can feel when someone is suffering and going through difficult moments	1	2	3	4
31	I can see the suffering of another person in their body language	1	2	3	4
